# Procedures on Demand: Evaluating Pediatric Procedural Training With Video Education

**DOI:** 10.7759/cureus.100265

**Published:** 2025-12-28

**Authors:** Jennifer E Udeogu, Julia Klein, Shreya Raghavan, Jeff Louie

**Affiliations:** 1 Pediatric Emergency Medicine, Baylor College of Medicine/Texas Children's Hospital, Houston, USA; 2 Pediatrics, University of Minnesota/Masonic Children's Hospital, Minneapolis, USA; 3 Pediatrics, University of Minnesota, Minneapolis, USA; 4 Pediatric Emergency Medicine, University of Minnesota, Minneapolis, USA

**Keywords:** educational innovation, low-cost training tools, medical education technology, pediatric residency, procedural training, resident education, social media in medical education, video-based learning

## Abstract

Background and objective

Procedural competence is crucial in pediatric residency training, but many residents report few opportunities to develop hands-on skills. We launched a pilot initiative using curated instructional videos from social media to offer easily accessible, supplemental procedural education. The goal was to use these curated videos as a practical, cost-effective, and scalable way to supplement procedural education in pediatric residency training.

Methods

This pilot project ran from July 2023 to June 2024 within a single urban, academic pediatric residency program. High-quality procedural videos were collected from social media platforms, evaluated for clinical accuracy, and made available on a centralized website. Resident engagement was monitored using Google Analytics, and informal feedback on usability and perceived benefit was gathered.

Results

Over one year, the website received 377 views from 78 unique users, representing approximately 70% of the residency cohort. Pediatric emergency medicine (PEM) videos had the highest average engagement time, while neonatal intensive care unit (NICU) videos achieved the most views per user. Residents reported that the videos were easy to access and useful for building procedural confidence. Challenges included occasional video removals, which were resolved through periodic content review.

Conclusions

This initiative demonstrates the feasibility of using curated social media-based videos as a cost-effective, scalable educational supplement to enhance pediatric procedural training. While early engagement data are encouraging, further research is needed to assess their direct impact on procedural competency and patient outcomes.

## Introduction

The Accreditation Council for Graduate Medical Education (ACGME) requires procedural competence to be an essential part of medical residency training, allowing graduating residents to function as independent physicians who can perform procedures and provide high-quality care to patients within their scope of practice. Over the past decade, various studies have explored methods to enhance procedural skills, especially among pediatric residents. Most literature indicates that simulation-based training is a promising approach to bridge the gap between theory and practice [1,2]. Simulation-based training provides curated, risk-free scenarios for residents to learn and practice procedures. However, common challenges remain, including high costs, significant resource demands, incomplete replication of clinical complexity, and difficulties in seamlessly integrating these sessions into existing curricula [3,4].

At our institution, residents expressed a desire for further education on procedures to increase familiarity and confidence with these skills, but they also face time constraints due to clinical responsibilities, didactic learning, and duty hour limitations. To address the gap between theoretical knowledge and practical skills for pediatric residents, we developed an initiative to pilot the feasibility of incorporating easily accessible, concise training videos from social media to enhance procedural skills while improving simulation-based training and its integration. Embedding social media videos into medical education has gained significant attention in recent years, particularly after the COVID-19 pandemic [5,6]. Social media is a relatively new and emerging field in graduate medical education, offering an opportunity as an alternative method for delivering succinct and tangible educational content [7]. Consequently, we created a database of screened and reviewed videos from social media platforms with the potential for subsequent studies on resident engagement and its effect on procedural competency. This study aims to describe our process and demonstrate the initial impact and feasibility of the pilot initiative, particularly regarding resident engagement.

## Materials and methods

We conducted a mixed-methods study to evaluate the impact of social media-based instructional videos on pediatric procedural training. This pilot phase of the study focused primarily on the quantitative component, tracking user engagement with the video repository. In addition to the quantitative data, we collected informal qualitative feedback from pediatric residents to provide insights into their experiences with the intervention.

Setting and participants

We implemented this educational intervention as a supplemental resource in the pediatric residency program at a single urban academic medical center from July 1, 2023, to June 30, 2024. The target learners were pediatric residents across all postgraduate years (PGY 1-4, including Medicine/Pediatrics residents). The primary aim of these educational videos was to improve procedural competency among pediatric residents by creating a centralized repository of high-quality instructional videos demonstrating commonly performed pediatric procedures. We obtained an IRB waiver from our institution confirming that the project did not require IRB review or approval.

Intervention development

A multidisciplinary team, including attending physicians, residents, and administrative staff from the emergency department, collaboratively designed and implemented the educational intervention. We sourced instructional videos from publicly available social media platforms such as TikTok, YouTube, and the established educational resource NeoReviews [8]. We prioritized selection based on clarity and medical accuracy, with a target video length of under fifteen minutes to facilitate efficient learning. All videos from YouTube and TikTok were vetted by attending physicians who routinely perform the depicted procedures. The emergency medicine attending physician responsible for overseeing educational resources selected and reviewed videos likely to be encountered by residents during rotations in the emergency department, pediatric intensive care unit, and neonatal intensive care unit. We excluded videos that did not meet these criteria. Videos from NeoReviews were not screened because they are an educational resource approved by the AAP [8]. The overall review process assessed clinical accuracy, safety, relevance, and adherence to procedural guidelines.

Implementation

Our team curated selected videos and linked them to a centralized website hosted on Google Sites (Appendix A). To ensure point-of-care access, we made the website accessible via QR codes strategically placed in the pediatric emergency department, where most procedures occur. This strategy is effective in promoting the use of educational resources [9]. Awareness of the educational intervention was enhanced through weekly emails to residents and updates to attending physicians working in the emergency department, neonatal intensive care unit, and pediatric intensive care unit. The implementation required minimal faculty time beyond video vetting and did not involve structured training sessions. The intervention incurred no additional financial costs.

Evaluation strategy

We assessed engagement using Google Analytics to track the website's activity and overall effectiveness. When a user interacts with the website, several variables are recorded: total views, active users, and views per active user. Total views represent the total number of times the webpage was accessed, including repeat views by the same user. Active users are defined as the number of users engaged on the website for more than 10 seconds or who navigate more than one page within a given time period. Views per active user indicate the number of views by each unique user. Monitoring these variables allowed us to better understand viewership and evaluate the effectiveness of our project. To ensure content optimization, residents were encouraged to provide feedback on the usability and perceived effectiveness of the videos. Descriptive statistics were used to analyze the data. We calculated means, ranges, and standard deviations (SD) across procedural categories (pediatric emergency medicine (PEM), neonatal intensive care unit (NICU), pediatric intensive care unit (PICU) to summarize engagement metrics. These included mean views, mean active users, average views per user, and mean engagement time.

Cost, efficiency, and user experience 

The project is cost-free, utilizing publicly available instructional and social media videos and requiring no additional expenditure for materials, faculty training, or simulation resources. Implementation was time-efficient, as faculty oversight was limited to reviewing videos for accuracy, and no structured training sessions were required. The centralized website is hosted on the free Google Sites platform, and resident engagement was easily tracked using Google Analytics. From an acceptability perspective, informal feedback from residents indicated that the intervention was easy to use, accessible at the point of care, and helpful in boosting procedural confidence, highlighting its practical value in a time-constrained learning environment.

## Results

During the first full academic year of implementation, we observed substantial engagement with the video-based procedural training website. At our institution, the intended audience and sample size, consisting of categorical pediatrics residents and medicine-pediatrics residents, were estimated to include 110 residents. Using Google Analytics, we tracked three key engagement metrics: total views, number of active users, and average engagement time. Over the first year, the website recorded a total of 377 views from 78 unique users, yielding an average of 4.83 views per user. Engagement varied by procedural category, as expected.

As shown in Table 1, the homepage had the highest number of views, with 224 views from 76 active users, averaging 2.95 views per active user and a mean engagement time of 15 seconds. Among specialty categories, PEM procedures had 59 views from 32 users (1.84 views per user, 25-second average engagement), illustrating the longest average engagement time. NICU procedures had 50 views from 19 users (2.63 views per user, 13-second engagement), illustrating a shorter engagement time but yielding a higher mean number of views per user, and PICU procedures had 44 views from 25 users (1.76 views per user, 14-second engagement).

**Table 1:** Procedure education website engagement metrics PEM: pediatric emergency medicine; NICU: neonatal intensive care unit; PICU: pediatric intensive care unit

Procedure type	Views	Active users	Views per active user	Average engagement time per active user
General pediatric procedure education (homepage)	224	76	2.95	15s
PEM procedures	59	32	1.84	25s
NICU procedures	50	19	2.63	13s
PICU procedures	44	25	1.76	14s

These patterns suggest that while PEM content garnered the most consistent engagement, NICU videos were more frequently revisited by individual users, potentially indicating higher utility for preparing for infrequently performed procedures. When aggregated across categories, PEM, NICU, and PICU videos had a mean of 51.0 views (range: 44 - 59; SD = 7.6), 25.3 active users (range: 19 - 32; SD = 6.8), 2.08 views per active user (range: 1.76 - 2.63; SD = 0.48), and an average engagement time of 17.3 seconds (range: 13 - 25; SD = 6.4), as shown in Table 2.

**Table 2:** Aggregated video engagement metrics across PEM, NICU, and PICU categories PEM: pediatric emergency medicine; NICU: neonatal intensive care unit; PICU: pediatric intensive care unit; SD: standard deviation

Metric	Mean	Range	SD
Views	51	44–59	7.6
Active users	25.3	19–32	6.8
Views per active user	2.08	1.76–2.63	0.48
Average engagement time (sec)	17.3	13–25	6.4

Importantly, the site showed a follow-through rate of 68.3%: of the users who visited the homepage (224 views), the combined specialty content (PEM, NICU, PICU) accounted for 153 views. This suggests that over two-thirds of users moved beyond the landing page to engage with targeted educational materials. Finally, informal resident feedback indicated the videos were helpful as a just-in-time resource to prepare for procedures in clinical settings. Although formal skill or knowledge assessments were not conducted in this pilot phase, the usage metrics and rewatch rates support the feasibility and perceived value of this educational approach.

## Discussion

This pilot project introduces an innovative approach to residency procedural education by leveraging curated social media videos that are accessible at the point of care. Unlike traditional simulation-based learning, which often requires substantial institutional resources, our method offers a cost-effective alternative that integrates seamlessly into existing resident workflows and aligns with modern digital learning habits. The integration of social media into graduate medical education, when guided by robust content review for accuracy and clinical relevance, represents an evidence-based tool for procedural training. This approach provides residents with accessible, repeatable video content that extends beyond the traditional “see one, do one” model and addresses the limited procedural exposure typical in pediatric training [10,11]. Prior studies on e-learning, mobile applications, and video-based instruction in medical education have shown promising results, particularly in improving knowledge retention and learner satisfaction [12]. However, few interventions have specifically focused on integrating curated social media content into clinical workflows, positioning this project as a novel contribution to the evolving field of digital medical education.

Though early engagement metrics are encouraging, we did not directly assess procedural competence or clinical outcomes. This limitation restricts the ability to draw definitive conclusions about educational effectiveness beyond self-reported confidence. Future work should incorporate objective assessments, such as pre- and post-intervention knowledge testing, procedural checklists, or supervisor evaluations, to better understand the intervention’s impact on skill acquisition and patient outcomes [13]. Sustainability and long-term scalability remain key challenges. Our intervention relied on publicly available content, making it vulnerable to occasional video removals or account changes. Further efforts could explore collaborations with professional societies, such as the American Academy of Pediatrics, or the development of institutional content to ensure content stability, as demonstrated in the study by Srinivasa et al. [6], and to support ongoing quality assurance. Moreover, expanding this model to include specialty-specific procedural libraries or interprofessional content could further enhance its reach and educational value. We hope to continue this project and measure its impact on procedural competency across various training levels while continuously improving the website through resident and faculty feedback.

Our project has several limitations. While approximately 70% of the viewership is estimated to represent the intended audience, pediatric residents, Google Analytics does not allow for precise user identification. Consequently, total view counts may also include engagement from medical students, attending physicians, and other institutional staff. This constraint limits our ability to attribute engagement metrics solely to residents. Another limitation was the occasional unavailability of linked videos. This issue arises when videos are removed due to account discontinuation or social media platform policies that restrict medical procedure content. To mitigate this, members of the education team regularly check that the links on the website are functional.

While this project was conducted at a single site, its low-cost, easily replicable design suggests it could be implemented across other residency programs and specialties, particularly in resource-constrained or rural settings where simulation resources are limited [3]. As medical education continues to evolve, thoughtfully integrating digital tools and social media platforms presents a valuable opportunity to enhance learning experiences. We hope to expand our vision to include residency-specific procedural videos outside of pediatrics to improve patient care outcomes across the institution. A potential future direction includes adapting the project into an app-based format to increase accessibility. The literature review indicates that these tools serve as effective teaching resources and support the retention of knowledge and skills [14,15].

## Conclusions

This educational initiative demonstrates that integrating curated, social media-based videos into pediatric residency training could be a feasible, cost-effective, and scalable approach to supplement procedural education, as implemented at our institution, and primarily supports feasibility, resident engagement, and perceived educational value. By providing residents with an easily accessible, curated repository of high-quality videos, we addressed challenges related to time constraints and limited procedural opportunities within the residency curriculum, with the goal that the videos would serve as supplemental educational resources before procedures or during simulation training. While initial engagement metrics and qualitative feedback are promising, we acknowledge that further research is essential to formally assess their impact on skill acquisition and clinical outcomes. Expanding this model across other specialties and institutions could provide broader benefits to medical education, highlighting the need for responsible, quality-assured use of digital platforms in shaping future healthcare training and ultimately enhancing patient care.

## Figures and Tables

**Figure 1:**
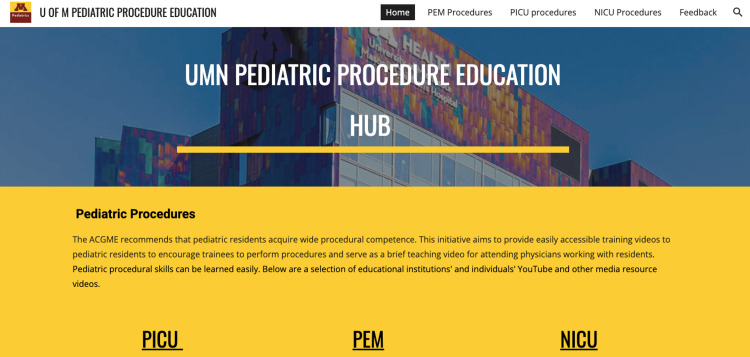
Screenshot of centralized procedural video website

## Appendices

Appendix A

Centralized Procedural Video Website - Screenshot and Structure
